# Circular RNAs in stem cell differentiation: a sponge-like role for miRNAs

**DOI:** 10.7150/ijms.56457

**Published:** 2021-04-22

**Authors:** Jian Zhou, Cheng Qiu, Zhihua Fan, Tianyi Liu, Tang Liu

**Affiliations:** 1 Department of Orthopedics, The Second Xiangya Hospital, Central South University, Changsha, Hunan, 410011, P. R. China.; 2 Department of Orthopaedic Surgery, Qilu Hospital, Cheeloo College of Medicine, Shandong University, Jinan, Shandong, 250012, P. R. China.; 3 Cheeloo College of Medicine, Shandong University, Jinan, Shandong, 250012, P. R. China.; 4 Xiangya School of Medicine, Central South University, Changsha, Hunan, 410013, P. R. China.; 5 Department of Dermatology, Xiangya Hospital, Central South University, Changsha, Hunan, 410008, P. R. China

**Keywords:** CircRNA, stem cell differentiation, miRNA, sponges, ceRNA

## Abstract

Circular RNAs (circRNAs) are novel endogenous non-coding RNAs that play a critical role during cellular signal transduction, gene transcription and translation. With the rapid advancement of bioinformatics analysis tools and high-throughput RNA sequencing, numerous circRNAs with important biological features have been identified. They function as competing endogenous RNAs (ceRNAs) of microRNAs and as such exhibit the potential to act as biomarkers for stem cell differentiation. In the recent past, several studies have shown the involvement of circRNAs in stem cells differentiation. The present review summarizes the molecular characteristics, biogenesis and mechanisms of newly identified circRNAs in the differentiation of stem cells. In conclusion, circRNAs regulate the stem cells differentiation *via* their ambient binding efficacy to modulate miRNA expression, as well as related gene translation. We believe that this review will provide reference guidance for future studies on stem cell differentiation.

## Introduction

Circular RNAs (CircRNAs) used to be misjudged as irrelevant during DNA transcription. However, with the development of RNA sequencing technology, circRNAs received increased attention 1-3. Studies have shown that circRNAs are stable, abundant, cell-type specific and evolutionarily conserved 4, 5. Some circRNAs can increase the complexity of RNA regulatory networks by acting as microRNA (miRNA) sponges, thereby suppressing the function of miRNAs in eukaryotic cells 3, 6-8. There are other functions of circRNAs which include: 1) protein sponges that bind some proteins preventing them from binding to other targets 9; 2) protein scaffolding which harbors binding sites for enzymes and substrates to facilitate their contact 10; 3) splicing and transcription supporter that function in the nucleus to promote transcription or alternative splicing 1, and 4) translated into protein 11. Among these functions mentioned above, the function of circRNAs as miRNA sponges is the most universal and most valued. The miRNAs regulate a battery of biological processes by binding to the 3'-untranslated regions (3'-UTR) of target messenger RNAs (mRNAs) to arrest their translation 12. This form is mainly presented by promoting mRNA degradation *via* polyA tail shortening 13. To inhibit the functions of miRNAs, antisense oligonucleotides or antagomirs were commonly used *in vitro* or *in vivo* 14, 15. Recently, the theoretic hypothesis proposes that circRNAs bind to miRNAs to serve as competitive inhibitors and are thus referred as “miRNA sponges” that interrupt subsequent biological functions 2, 5. The binding sites of circRNAs for miRNA exist either in the 3′-UTR or in the non-coding transcript of a specific gene 16. Thousands of circRNAs have been predicted and identified by bioinformatics analysis and high throughput RNA sequencing in human genome. These studies have also indicated that circRNAs may serve as important post-transcriptional regulators 17, 18. Recently, circRNAs downregulating the miRNA expression acting as miRNA sponges have been investigated in several different stem cells 19-22. Cell differentiation causes the cells to change from a non-specific (non-specialized) state to a morphologically and functionally specific (specialized) state. This leads to the development of different types of cells with different phenotypic structures, and phenotypes which consequently form different tissues, organs, and systems to perform the complex functions of the body. During cell differentiation, totipotent or pluripotent stem cells gradually differentiate into mature cells and subsequently acquire a specific function, in which fluctuating expression of different genes play a vital role. In recent years, circRNAs have been more and more valued for their regulatory function on gene expression. A subset of circRNAs has been found to be enhanced in naive human ESCs (hESCs), for instance, circBIRC6 which acts as a sponge both for miR-34a and miR-145, involved in curbing the hESCs differentiation 22. Additionally, during the differentiation of stem cells into mature cells, such as myocardial differentiation, epithelial differentiation, neuronal differentiation and osteogenic differentiation, the expression of some of the circRNAs have been found to remarkably elevated and many of these were derived from developmental genes 22-25.

Collectively, a large number of studies have revealed a sponge-like role for circRNAs, which prevents the expression of target miRNA consequently regulating the activity of downstream genes 23, 24. Herein, we review the biogenesis, characterization and classification of circRNA and explore the relationship between miRNA expression and circRNA in stem cells differentiation.

## Biogenesis of circRNAs

CircRNAs differ from other RNA species in that the 3' and 5' ends are covalently linked. Typical splicing signals are located on both sides of the end junctions of circRNAs indicative of the involvement of the splicing bodies in the generation of circRNAs and in constitutive and selective linear RNA splicing 25. CircRNAs are formed by back-splicing 26. According to a previous study, there are 6 models that explain the formation of circRNAs; A. cyclization driven by tRNA splicing 27, B. direct cyclization of lariat introns 26, C. cyclization mediated by RNA-binding proteins (RBPs) 28, D. cyclization driven by lariat mechanism 26, E. cyclization mediated by intron pairing 1, 26, F. cyclization driven by rRNA splicing 29.

## CircRNAs as sponges of miRNAs in stem cells

**Figure 1:**
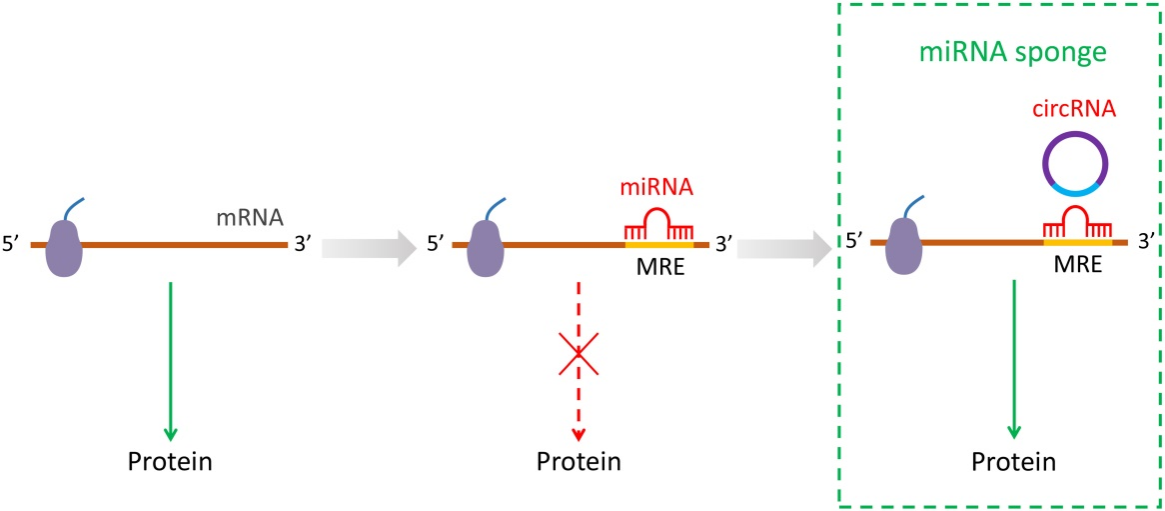
The miRNA sponging by circRNA during post-transcriptional process.

The most significant role of circRNAs is that circRNAs can repress the function of miRNAs by binding them as competing endogenous RNAs (ceRNAs) 16, 26, 30. It has been shown that circRNAs have multiple miRNA response elements (MREs) 3. A previous study has identified over 60 conserved seed match segments on miR-7 for circRNAs sponge suggestive of a very dense combination 3. The interactions between circRNAs with miRNAs by sponges are recognized gradually during cellular living activities 23, 24. The function of circRNAs is dependent on the cellular localization. CircRNAs turn into large RNA-protein complexes by binding to RBPs in the nucleus, which in turn may affect the transcription of mRNAs 31, 32. Moreover, circRNAs also exhibit regulatory role in gene expression through linear pre-mRNA splicing competition. For example, circMbl sponges to muscle blind (MBL) by regulating the splicing of its own pre-mRNA into a translatable mRNA or circMbl 33. It is known that circRNAs can participate in many biological processes, while many functions remain yet to be elucidated. Almost all known circRNAs display sponge activity that localize to the cytoplasm 16. Hitherto, accumulating evidence suggest that circRNAs regulate the development and progression of stem cells differentiation by suppressing miRNA species (**Figure 1**).

## Identified circRNAs in stem cells differentiation

Recent evidence has shown that circRNAs are associated with the differentiation of several types of stem cells, such as dental pulp stem cells (DPSCs), maxillary sinus membrane stem cells (MSMSCs), periodontal ligament stem cells (PDLSCs), embryonic stem cells (ESCs), bone marrow-derived mesenchymal stem cells (BMSCs), induced pluripotent stem cells (iPSCs), epidermal stem cells (EpSCs), germline stem cells (GSCs), intestinal stem cells (ISCs) and hematopoietic stem cells (HSCs).

## CircRNAs in the differentiation of dental pulp stem cells

**Figure 2:**
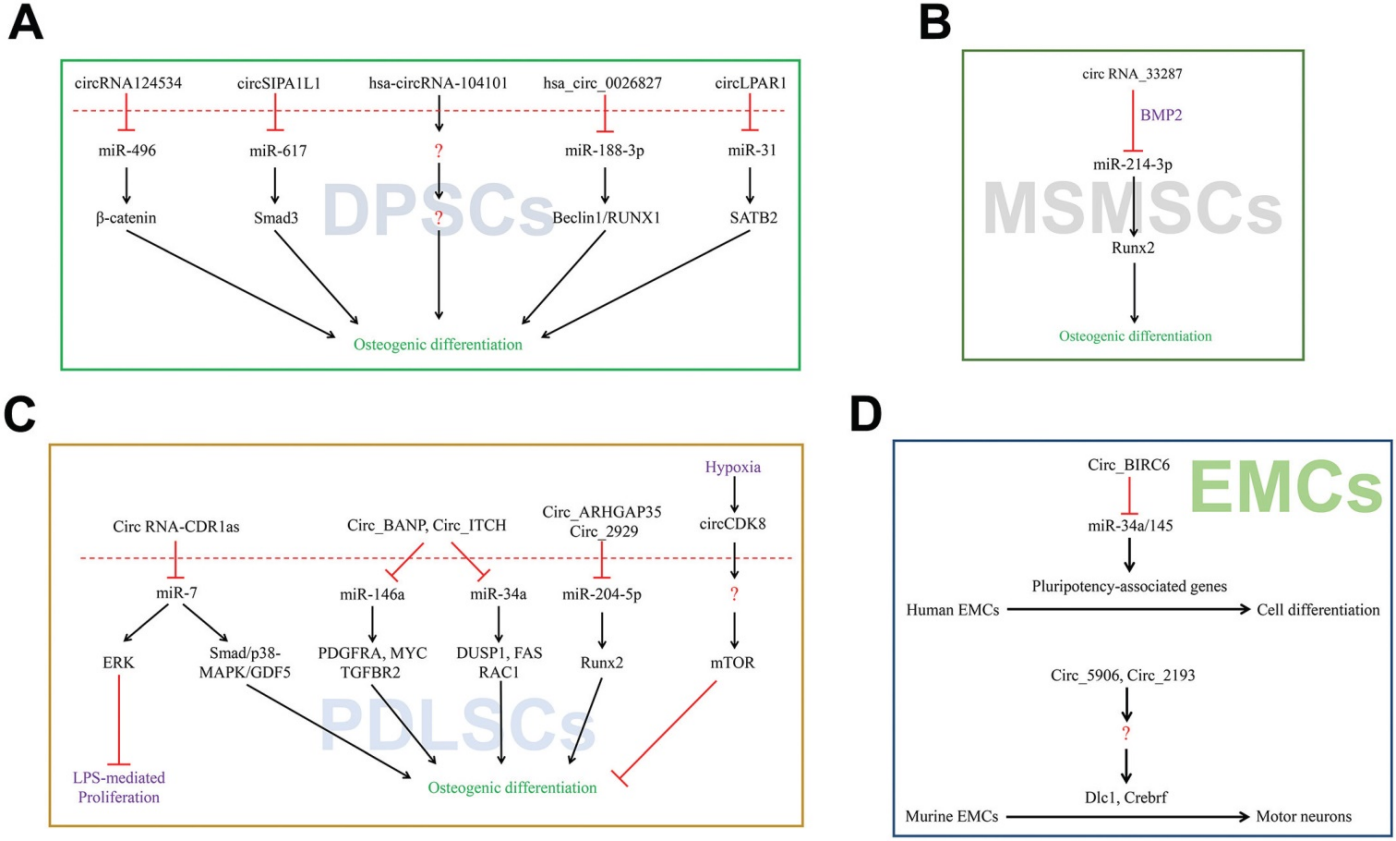
CircRNAs associated with stem cells differentiation. (A-D) CircRNAs affected its target miRNAs *via* miRNA sponge which reduce the function of translation in DPSCs, MSMSCs, PDLSCs and EMCs.

Dental pulp stem cells (DPSCs) are present in the dental pulp and exhibit higher proliferation rates, self-renewal and cloning potential due to its vested abilities. Recent studies have identified that circRNAs play a critical role in the involvement in the differentiation of DPSCs. From these investigations, circRNA124534 has been reported to promote osteogenic differentiation as a miR-496 sponge in human DPSCs. Additionally, circRNA124534/miR-496/β-catenin axis has been demonstrated as a covert curative clue for elevation of dental bone remodeling 34. Moreover, circRNA hsa_circ_0026827 has also been reported to play a sponging role by targeting miR-188-3p *via* Beclin1 and RUNX1 signaling pathways that enhances the osteogenic differentiation in human DPSCs 35. In yet another study, circRNA SIPA1L1 was found to regulate miR-617/Smad3 axis to potentiate osteogenic differentiation of DPSCs 20. Furthermore, role of exosomes in osteogenic differentiation of DPSCs has been investigated and it was found that circLPAR1 adsorbs to hsa-miR-31 to avoid its inhibitory effects on osteogenesis of DPSCs 36. A microarray analysis revealed 43 upregulated circRNAs and 144 downregulated circRNAs to be differentially expressed in human DPSCs during odontogenic differentiation process. Moreover, hsa_circRNA_104101 was highly assumed to enhance the odontogenic differentiation of human DPSCs 37. Based on above, it is well established that the common mechanisms of circRNAs in DPSCs are competently binding with miRNAs to deliver a promoting effect on osteogenic differentiation (**Figure 2A**).

## CircRNA in maxillary sinus membrane stem cells differentiation

Maxillary sinus membrane stem cells (MSMSCs) exhibit osteogenic potential and may find their use in bone repair of maxilla. By using microarray analysis, Peng et al. revealed that the novel pathway circRNA_33287/miR-214-3p/Runx3 plays a role in the regulation of the osteoblastic differentiation in MSMSCs. Additionally, BMP2 may also induce the osteogenic differentiation by enhancing the expression of circRNA_33287 38. However, only a few studies have been carried out on this aspect of circRNAs in MSMSCs and therefore the underlying mechanisms remain largely elusive (**Figure 2B**).

## CircRNAs in periodontal ligament stem cells osteogenic differentiation

Gu et al. indicated that in the phase of periodontal ligament stem cells (PDLSCs) osteogenic differentiation, recognition and disposal of circRNAs plays an imperative role in ceRNAs. They used RNA sequencing and found 1456 circRNAs to be differentially expressed in normal and osteogenic inductive PDLSCs. Furthermore, 1382 circRNAs were found to combine with 148 common miRNAs, which were predicted to be involved in osteoblast differentiation 39. By acting as miR-7 sponge, circRNA-CDR1as has been reported to mediate the inhibitory effect of LPS on proliferation of PDLSCs by activating the ERK signaling pathway 39. Importantly, circRNA-CDR1as was also reported to regulate the osteoblastic differentiation of PDLSCs by triggering the activation of Smad and p38 MAPK signaling pathway, as well as upregulation of GDF5 40. The circRNA-CDR1as/miR-7 axis may be involved in PDLSCs upon periodontitis burst. Moreover, Zheng et al. ascertained that hsa_circ_0003489 is located at cyclin-dependent kinase 8 (CDK8) gene so they referred it as circCDK8 41. Meanwhile, the overexpression of circCDK8 was found to induce autophagy and apoptosis, whereas the silencing of circCDK8 reversed the inhibitory effects of cobalt chloride (CoCl_2_) on osteogenic differentiation of PDLSCs. Hereof, Zheng et al, pointed out that circCDK8 inhibits the osteogenic differentiation of PDLSCs in a hypoxic environment 42. In addition, the mechanical force may induce the changes in the expression of circRNAs in PDLSCs with regard to miRNAs transformation 42. A previous study showed that miRNA146a exerted its unique role by post-transcriptionally modulating the expression levels of the target genes to regulate the PDLSC osteogenic differentiation 43. Moreover, in another study Chen et al. 44 showed that miRNA-34a inhibits the differentiation of osteoblasts. Interfering with miRNA34a and miRNA146a as we inferred, circRNA BANP coupled with circRNA ITCH could modulate PDLSC osteogenic differentiation through MAPK pathway. DUSP1, FAS and RAC1 are targeted by miRNA34a, while as PDGFRA, TGFBR2 and MYC are targeted by miRNA146a. Kovacić et al. showed that Fas/Fas ligand system inhibits the differentiation of murine osteoblasts 45. The inhibition of RAC1 has been reported to promote the BMP-2-induced osteoblastic differentiation 46. Circ_BANP/circ_ITCH-miR-34a-FAS/RAC1 may be an axial regulator of osteoblast differentiation. Zheng et al examined the expression profile of circRNAs, miRNAs and mRNAs during the osteogenic differentiation of PDLSCs, and a network containing potential targeting relationships was constructed 47. A previous report indicated that miR-204 inhibits the osteogenesis and promotes adipogenesis of mesenchymal stem cells by targeting Runx2 48. In the regulatory aspect of osteogenesis and chondrogenesis, RUNX2 has been regarded as a pivotal transcription factor 49. Circ_2929 and circ_ARHGAP35-miR-204-5p-Runx2 may be potential axial regulators of osteoblast differentiation (**Figure 2C**).

## CircRNAs in the differentiation of embryonic stem cells

Using RNA-sequencing analysis, a total of 3,894 circRNAs within the body of 2,097 known genes were identified expressed in murine embryonic stem cells (ESCs) derived motor neurons (MNs) 50. Analysis from studies showed that most of them having a homologous trend like the annular structures, but the circ_5906 and circ_2193 were counter examples. According to Lorenzo Errichelli et al reported that the expression of circ_5906 upregulates during the mESCs differentiation, whereas circ_2193 was found to be downregulated 50. Interestingly, the RNA-binding protein FUS-dependent linear correlations were highly presented in mESCs and down-regulated under differentiation. Both of them may play an important role in preventing the murine embryonic stem cell differentiation. Intriguingly, several candidate circRNAs were identified to play crucial roles in neuronal differentiation in mESCs and circZNF827 was reported to be negatively regulated in this process through nerve growth factor (NGF) signaling 51.

Human embryonic stem cells (hESCs) with the characteristics of unlimited self-renewal and pluripotency are studies on first priority for applicability 52. It is known that miR-34a and miR-145 can promote *in vitro* differentiation of hESCs by repressing pluripotency-associated genes 22. Yu et al. identified a subgroup of circRNAs that are enriched in undifferentiated hESCs 22. Additionally, circBIRC6 is associated with the pluripotent state. Moreover, it was found that circBIRC6 interacts with miR-34a and miR-145 and is enriched in the AGO2 complex. Correspondingly, circBIRC6 suppresses hESCs differentiation by down-regulating the expression of miR-34a and miR-145. Furthermore, the biogenesis of circBIRC6 in hESCs was found to be regulated by the hESC-enriched splicing factors (SFs) ESRP1. The expression of ESRP1 was controlled by the core pluripotency-associated factors, NANOG and OCT4. CircRNAs can suppress the hESCs differentiation by serving as a microRNA “sponge” (**Figure 2D**).

## CircRNAs in bone marrow-derived mesenchymal stem cells osteoblast differentiation

**Figure 3:**
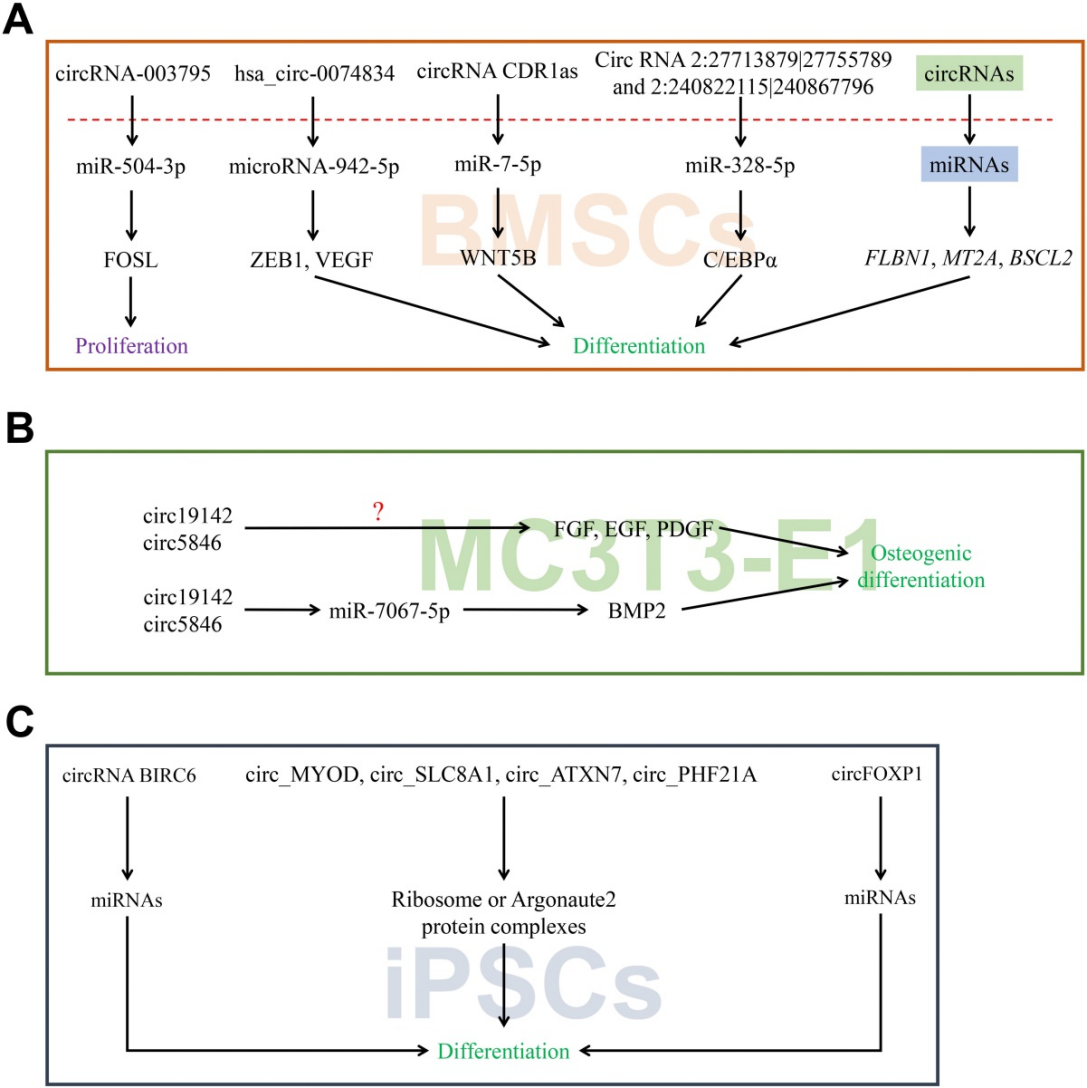
CircRNAs associated with stem cells differentiation. (A-C) CircRNAs affected its target miRNAs via miRNA sponge which reduce the function of translation in BMSCs, MC3T3-E1 and iPSCs.

The proliferation and differentiation of bone marrow-derived mesenchymal stem cells (BMSCs) into osteoblasts is first stage for osteogenesis. In presence of calcitonin gene-related peptide (CGRP) stimulation, the expression of circRNA_003795 was significantly increased to promote proliferation and FOS like 2 AP-1 transcription factor subunit (FOSL2) expression, whereas slicing it dramatically up-regulated the miR-504-3p expression. These results indicated that circRNA_003795 indirectly regulates FOSL2 expression *via* sponging of miR-504-3p to promote BMSCs proliferation which prepared for further differentiation 53. The milieu of BMSCs was also found to regulate its differentiation by influencing the expression of numerous circRNAs. As ceRNAs, circRNAs have been detected to exhibit variable expression in BMSCs on the different materials surface 54. Recently, our study also discovered hsa_circ_0074834 to act as a ceRNA to regulate the expression of ZEB1 and VEGF via microRNA-942-5p to promote osteogenic differentiation of BMSCs which is advantageous to bone remodeling 55. Opposite regulation of circRNAs with miRNAs was reported in the early osteogenic differentiation of BMSCs, and masked genes (*FLBN1*, *MT2A*, and *BSCL2*) were given rise to attention during this part 56. The adipogenic or osteogenic differentiation of BMSCs has been reported to be associated with the pathogenesis of steroid-induced osteonecrosis in femoral head. A recently carried out study identified circRNA CDR1as promotes the development of adipogenic differentiation which dampens the osteogenic differentiation in BMSCs via CDR1as-miR-7-5p-WNT5B axis providing new insights into the treatment of related diseases (**Figure 3A**) 57.

Previous studies showed that ERβ plays an important role in the osteogenesis and trigger MC3T3-E1 cells to undergo osteogenic differentiation 58. However, little is known about the relationship between circRNAs and bone metabolism. In a recent study, RNA-Seq was performed to detect the differentially expressed circRNAs between control and estrogen receptor beta (ERβ) deficient rat BMSCs. It was found that up to 146 cases of circRNAs were diversely expressed by the range of fold-change 2.0 (p ≤ 0.05). Moreover, a total of 68 circRNAs were down-regulated, while 78 were found to be up-regulated 59. Li et al constructed circRNA-microRNA network to predict the miRNA sponges. This study showed that ERβ may regulate osteoblast differentiation *via* miR-328-5p-mRNA axis. Previous study indicated that miR-328 up-regulates C/EBPα expression to inhibit cell proliferation 59. Taken together, circRNAs-miRNAs network plays an important role in the regulation of osteogenic differentiation and C/EBPα may be considered as a mediator during this process. RNA-seq was performed with the aim to explore differential expression of circRNAs in osteoblast differentiation using particular cells named MC3T3-E1 60. A total of 158 circRNAs were differentially expressed between BMP2-treated MC3T3-E1 cells and untreated control cells, and among them 74 were up-regulated while 84 were down-regulated. The expression of circRNA.5846, circRNA.19142 and circRNA.10042 was confirmed to be dramatically up-regulated in the BMP2-treated cells. BMP2 was found to enhance the bone formation and remodeling by regulating the transcription of osteogenic genes both in osteoblast and BMSCs. Bioinformatic analysis of circ_19142 and circ_5846 showed Ywhaz, Plcg1, Src, Thbs1, Ncor2, Rps6kb1, Pdk1, Xiap and Prkci are potential target genes related to FGF, EGF, PDGF and Wnt pathways during this process. The circ_19142/circ_5846-miR-7067-5p-mRNAs axis may participate in the regulation of osteoblast differentiation (**Figure 3B**).

## CircRNAs in induced pluripotent stem cells differentiation

Induced pluripotent stem cells (iPSCs) are the reversion of somatic cells differentiation and cell reprogramming. Several circRNAs have been shown to participate in this process. Yu et al reported that circRNA BIRC6 reprograms the somatic cells to human iPSCs by directly acting as a sponge for miRNA involved in the maintenance of the pluripotent state 22. Another circRNA, circFOXP1, has been shown to be downregulated in mesenchymal stem cells when reprogramming to pluripotency 61. The circular RNA map for human iPSCs of fetal origin was also provided and it may highly benefit from clinical research 62. Zhang et al demonstrated that circRNAs upregulate the expression levels of exogenous octamer-binding protein 4 (OCT4) by sponging regulatory microRNAs in iPSCs 63. Overall, circRNAs act as ceRNA for miRNAs that play a critical role during cellular reprogramming.

Previous studies have revealed a close linkage between alternations in ribosome and proliferation, cell development and differentiation. Siede et al used RNA-seq to detect 320 differentially expressed circRNAs in human dilated cardiomyopathy (DCM) and control patients 64. The same dynamics of circRNAs from ATXN10, CHD7, DNAJC6 and SLC8A1 were also validated. The rodent homologs of circ_MYOD, circ_SLC8A1, circ_ATXN7 and circ_PHF21A interact with the ribosome during human induced pluripotent stem cell derived cardiomyocytes (hiPSC-CMs) differentiation, which are highly relevant in cardiac dysfunction. Functional studies are required to further clarify the role of these circRNAs refer to their host genes in pathophysiology (**Figure 3C**).

## CircRNAs in epidermal stem cells differentiation

**Figure 4:**
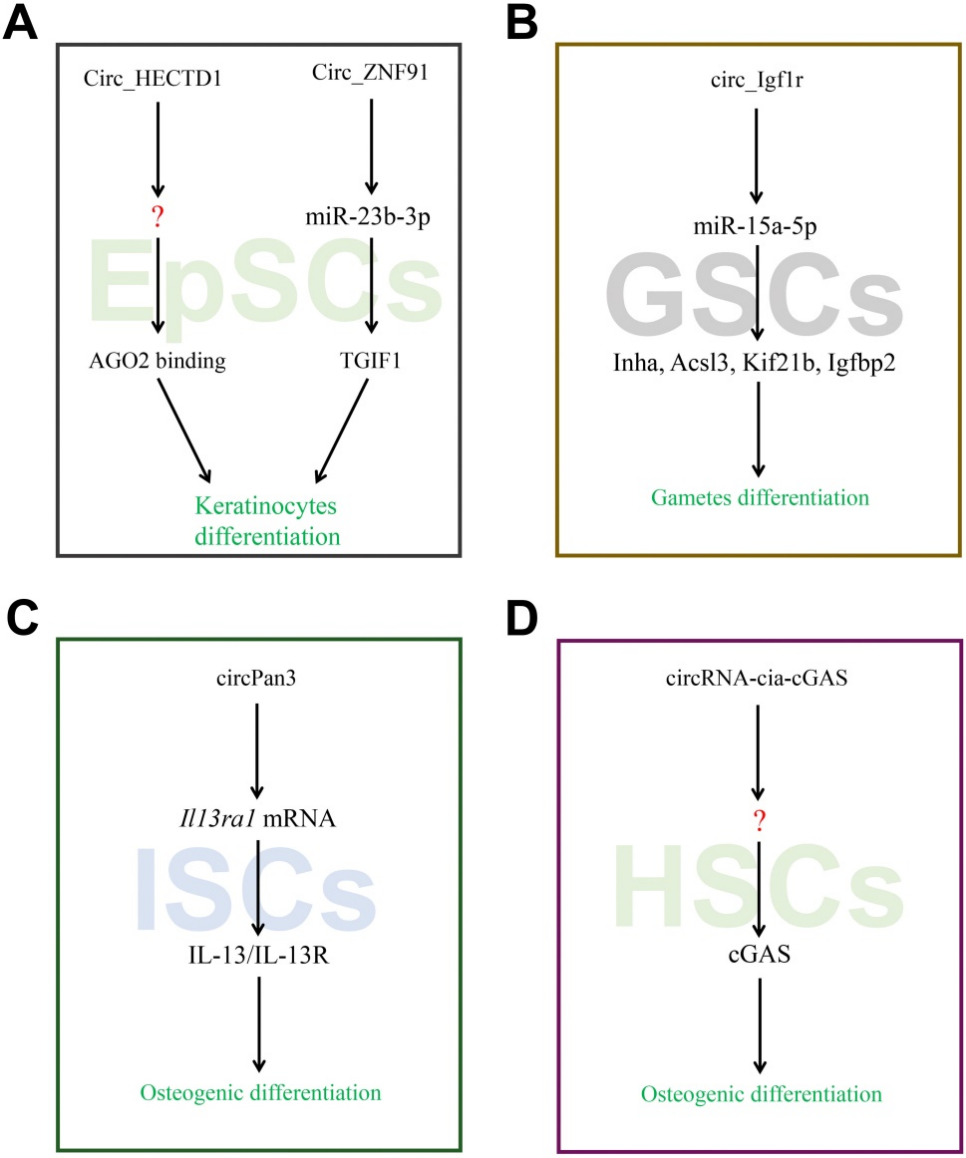
CircRNAs associated with stem cells differentiation. (A-D) CircRNAs affected its target miRNAs via miRNA sponge which reduce the function of translation in EpSCs, GSCs, ISCs and HSCs.

The RNA-seq was used to detect 624 unique circRNAs in the epidermal stem cells (EpSCs) and differentiated keratinocytes 65. It was found that the differentiated cells exhibit higher expression of circRNAs than the undifferentiated cells, many of which were derived from developmental genes. The discovered changes in circRNA expression were largely different from the expression of host gene. On the other hand, compared to stably expressed circRNAs, circRNAs which were up-regulated upon differentiation have stronger tendency to AGO2 binding and more promising miRNA combination sites. In particular, up--regulated circRNAs from the ZNF91 and HECTD1 genes have predicted miRNA target sites and exceptionally high numbers of AGO2 binding sites. Moreover, circ_ZNF91 contains 24 target sites for miR-23b-3p. It has been reported that circZNF91 functions as a miRNA sponge in human cells 65. Barbollat-Boutrand L et al. showed that mi-RNA-23b-3p regulates human keratinocyte differentiation via activation of the TGF-β-SMAD2 signaling pathway and repression of TGIF1 66. In yet another study, Circ_ZNF91-miR-23b-3p-TGIF1 axis has been reported to regulate the differentiation of EpSC (**Figure 4A**).

## CircRNA in germline stem cells differentiation

A study used high-throughput sequencing to identify the circRNAs in germline stem cells and the expression was further confirmed using RT-PCR 21, 67. Among them, 921 circRNAs showed sex-biased expression, which may reveal a vast amount of circRNA involved in diverse genetic imprinting between female and male germline stem cells. The circ_Igf1r can increase target gene Kif21b, Acsl3, Igfbp2 and Inha expressions by binding competitively with miRNA-15a-5p. A previous study indicated that circ_Igf1r acts as the ceRNAs of miR-15a-5p that targets the Kif21b, Acsl3, Igfbp2 and Inha mRNAs. Liu et al. showed that miRNA-15a-5p regulates VEGFA during endometrial mesenchymal stem cell differentiation. Kageyama A et al. identified ACSL3 as mediator of PA-induced osteoblastic differentiation 68. In another study, Xi et al. found that IGFBP-2 can directly stimulate osteoblast differentiation 69. Collectively, Circ_Igf1r-miRNA-15a-5p-Inha, Acsl3, Kif21b, and Igfbp2 may act as axis regulators of germline stem cells differentiation (**Figure 4B**).

## CircRNA in intestinal stem cells differentiation

Intestinal stem cells (ISCs) play a critical role in the repair of intestinal mucosal barrier by precise self-renewal and differentiation under the normal circumstances. Recent study showed that circRNA circPan3 is highly expressed in mouse and human ISCs. It binds to *Il13ra1* mRNA in ISCs to promote their stability, and is necessary for the self-renewal and differentiation of ISCs via IL-13/IL-13R-mediated signaling pathway 70. However, it is still unknown whether the circRNAs/miRNAs interactions exist during this process (**Figure 4C**).

## CircRNA in hematopoietic stem cells differentiation

Hematopoietic stem cells (HSCs) located in the bone marrow are adult stem cells of the blood system which exhibit the ability of long-term self-renewal and the potential to differentiate into various mature blood cells. Its aberrant form has been linked to the occurrence of hematological cancers. Benoit P Nicolet et al examined the expression pattern of circRNAs in human HSCs and found that circRNAs expression is cell-type specific as well as circRNAs splicing variants, and increase when cell maturates 71. Previously, Xia et al reported that a circRNA cia-cGAS to be highly expressed in the nucleus of long-term HSCs which binds to cGAS and subsequently inactivates the enzymatic activity of cGAS to protect dormant long-term HSCs from cGAS-mediated recognition, autoimmune response and exhaustion 72. Overall, the miraculous expression pattern of circRNAs in HSCs may manifest the vital role during hematopoiesis (**Figure 4D**).

## Discussion

Recently, circRNAs and stem cell have emerged as a new filed of research. Previous studies reported various methods to investigate the functions of circRNAs. Gene knockdown strategies can be used for functional study. We can identify target circRNAs *via* RNA sequencing and microarray. Quantitative real-time PCR, western blot, FISH and northern blot can be used to detect circRNA expression. Bioinformatics, immunohistochemical, and over-expression studies can be used to decipher the mechanisms. Furthermore, there are several databases or online services have been developed to provide the prediction or basic information about circRNAs and their potential regulatory networks (**Table 1**) 6, 73-80.

However, the research of circRNAs in stem cells differentiation is still in the infancy. CircRNAs used to be considered as errors in RNA splicing. However, they are now regarded as stable, abundant, active and ubiquitously expressed RNA molecules. CircRNAs play a remarkable role in translation, act as sponge and regulatory molecules and also may be utilized as reliable biomarkers. Meanwhile, circRNAs usually regulate miRNA expression *via* harboring MREs 81. The interaction of circRNA-miRNA can act through down-regulation transcriptional activity of miRNA target genes. Among them many miRNA target genes have been reported to play a significant role in stem cells differentiation, such as miR-34a and miR-146a. The miR-34a is the target gene of different circRNAs and plays an important role in the differentiation of hESCs and PDLSCs. In future, we can explore more connections between circRNAs and differentiation *via* those miRNAs. Gene silencing for this type of miRNAs may enable the achievement of the target and cause strong side effects in clinical applications. Nonetheless, the sponge effect of miRNAs on the target gene can be better targeted by specific circRNA. This point may provide a new way for cell experiments and animal models. The importance of circRNA dysregulation has been shown to be associated with the differentiation of different stem cells. In the present article we reviewed a novel mechanism by which circRNAs regulate miRNAs at the post-transcriptional level and we believe that it will provide insights into various processes associated with the stem cell differentiation. Additionally, the studies on miRNAs and circRNAs may enable the development of reliable diagnostic biomarkers and potential therapeutic targets for nonunion patients.

## Conclusions and perspectives

Taken together, circRNAs are confirmed to play a critical role in stem cell differentiation by regulation the miRNA functions via their sponging activity. The interactions between circRNAs and miRNAs may activate downstream signaling to promote stem cell differentiation. We hypothesize that whether circRNAs with potential sponging function is related to existence of each miRNAs. Furthermore, we conclude that the role of osteogenic promotion by circRNAs is the major part during the differentiation of several stem cells, such as DPSCs, MSMSCs, PDLSCs and BMSCs. Interventions targeting relevant circRNAs may have potential to regulate stem cells differentiation. Ultimately, we propose several regulatory axes such as circRNA_33287/miR-214-3p/Runx3, Circ_2929-circ_ARHGAP35/miR-204-5p/Runx2, circ_BANP/circ_ITCH-miR-34a-FAS/RAC1, and circ_19142/circ_5846-miR-7067-5p-mRNAs as vital players during stem cells osteogenic differentiation.

## Figures and Tables

**Table 1:** Online services of circRNA

Name	Website	Description
StarBase v2.0	http://starbase.sysu.edu.cn/	To predict miRNA-circRNA interactions
CircBase	http://www.circbase.org	To explore public circRNA datasets
Tissue-Specific CircRNA Database	http://gb.whu.edu.cn/TSCD/	To provide a global view for tissue-specific circRNA in main tissues of human and mouse
MiOncoCirc	https://mioncocirc.github.io/	Query the expression of a certain circRNA in different cancer clinical samples
ExoRBase	http://www.exoRBase.org	To trigger new circulating biomarker discovery and functional implication for human diseases.
CirclncRNAnet	http://app.cgu.edu.tw/circlnc/	To view the expression information of database samples according to gene names.
TRCirc	http://www.licpathway.net/TRCirc/view/index	To search and browse TFBSs of circRNAs, and other related information, for specific TFs, cell lines or circRNAs of interest
Circ2Traits	http://gyanxet-beta.com/circdb/	To collect circRNA databases that are potentially associated with human diseases or traits
CircRNADisease	http://cgga.org.cn:9091/circRNADisease/	To explore circRNA and disease associations
DeepBase v2.0	http://rna.sysu.edu.cn/deepBase/	To annotate and discover small (microRNAs), lncRNAs and circRNAs from next generation sequencing data
CircRNADb	http://reprod.njmu.edu.cn/circrnadb	A database for annotating exonic circRNAs, and can be a valuable resource for large-scale studies of circRNA
RegRNA 2.0	http://regrna2.mbc.nctu.edu.tw/index.html	To identify functional RNA motifs in an input RNA sequence
MiRWalk 3.0	http://mirwalk.umm.uni-heidelberg.de/	To predict and experimentally verify miRNA-target interactions with various novel and unique features
CircNet	http://circnet.mbc.nctu.edu.tw/	Identification of new circRNAs and integration of circRNA-miRNA-mRNA interaction network.
Cirbank	http://www.circbank.cn/help.html	To analyze the coding potential of circular RNA proteins
CircFunBase	http://bis.zju.edu.cn/CircFunBaseBlast/	To quickly query the name and function introduction of circleRNA
CircAtlas	http://circatlas.biols.ac.cn/	To use GO and KEGG databases to predict the potential functions of these circRNAs
BIOINF	http://www.bioinf.com.cn/	CircRNA primer design
Cancer-specific circRNA database	http://gb.whu.edu.cn/CSCD/	To find circRNA based on tumor cells
CIRCpedia	http://www.picb.ac.cn/rnomics/circpedia	To annotate alternative back-splicing and alternative splicing in circRNAs across different cell lines
Circular RNA Interactome	https://circinteractome.nia.nih.gov/	To predict and map the binding sites for RBPs and miRNAs on reported circRNAs

## Abbreviations

CircRNAs: Circular RNAs
DPSCs: dental pulp stem cells
ESCs: embryonic stem cells
BMSCs: bone marrow-derived mesenchymal stem cells
PDLSCs: periodontal ligament stem cells
PSCs: pluripotent stem cells
EpSCs: epidermal stem cells
GSCs: germline stem cells
ADSCs: adipose-derived mesenchymal stem cells
MSMSCs: maxillary sinus membrane stem cells
ISCs: intestinal stem cells
HSCs: hematopoietic stem cells
miRNAs: microRNAs
3'-UTR: 3ʹ-untranslated regions
mRNAs: messenger RNAs
RBPs: RNA-binding proteins
ceRNAs: competing endogenous RNAs
MREs: miRNA response elements
RPCs: RNA-protein complexes
MNs: motor neurons
NGF: nerve growth factor
SFs: splicing factors
CGRP: calcitonin gene-related peptide
FOSL2: FOS like 2 AP-1 transcription factor subunit
ERβ: estrogen receptor beta
CDK8: cyclin-dependent kinase 8
HIF-1α: hypoxia-inducible factor-1α
CoCl_2_: cobalt chloride
RNA-seq: RNA sequencing
DCM: dilated cardiomyopathy
hiPSC-CMs: human induced pluripotent stem cell derived cardiomyocytes
